# Factors Determining Postoperative Early Continence in Patients Undergoing Robotic Radical Prostatectomy

**DOI:** 10.3390/jcm14134405

**Published:** 2025-06-20

**Authors:** Metin Mod, Hasan Samet Güngör, Hakan Karaca, Ahmet Tahra, Resul Sobay, Abdurrahman İnkaya, Eyüp Veli Küçük

**Affiliations:** 1 Department of Urology, Basaksehir Cam Sakura City Hospital, 34480 Istanbul, Turkey; 2 Department of Urology, Health Science University Umraniye Training and Research Hospital, 34764 Istanbul, Turkey; drsametgngr@gmail.com (H.S.G.); hakankaraca1993@hotmail.com (H.K.); ahmettahra@gmail.com (A.T.); drresulsobay@gmail.com (R.S.); ainkaya@hotmail.com (A.İ.); eyupveli@hotmail.com (E.V.K.)

**Keywords:** prostate cancer, urinary incontinence, robotic surgery, multiparametric MRI

## Abstract

**Background/Objectives**: Prostate cancer is the second most common malignancy in men, and robot-assisted radical prostatectomy (RARP) has become a preferred treatment for localized disease. Postoperative urinary continence is a key determinant of quality of life. The aim of this study was to evaluate the preoperative patient characteristics and multiparametric magnetic resonance imaging (mpMRI) data that determine early postoperative continence in patients who underwent robotic radical prostatectomy at our clinic. **Methods**: In this study, patients who underwent robotic radical prostatectomy at our clinic between March 2020 and June 2022 were evaluated. The patients’ demographic data, preoperative PSA levels, digital rectal examination findings, preoperative lower urinary tract symptoms, sexual function, mpMRI findings, Briganti scores, surgical techniques used during the procedure and postoperative continence status were assessed. **Results**: A total of 111 patients were included in the study. The mean age of the patients was 61.1 years. The median follow-up duration was twelve months. According to the postoperative continence status, 22% of the patients were incontinent, 53% had moderate continence and 24% were fully continent in the first month. At the third month, 16.8% of the patients were incontinent, 31.3% had moderate continence and 51.8% were fully continent. At the one-year postoperative follow-up, the percentages of incontinent, moderately continent and fully continent patients were 4.8%, 13.2% and 81.9%, respectively. Urethral width in mpMRI (*p*: 0.012), pelvic transverse (*p*: 0.002) and AP (anterior–posterior) diameters (*p*: 0.033), preoperative IPSS scores (*p*: 0.033) and the presence of bilateral nerve-sparing surgery (*p*: 0.047) were found to be associated with postoperative urinary continence. No significant differences were found between groups regarding the relationship of other parameters evaluated by mpMRI with continence. **Conclusions**: In our study, preoperative IPSS scores, urethral width in mpMRI, pelvic transverse and AP diameters and the performance of nerve-sparing surgery were associated with early postoperative continence. Further studies with larger patient populations are needed to better understand the long-term predictors of postoperative urinary incontinence following radical prostatectomy.

## 1. Introduction

Prostate cancer (PCa) is the second most commonly diagnosed malignancy and the fifth leading cause of death in men. Every year, 1.4 million men are diagnosed with prostate cancer and 375,000 men die from prostate cancer annually. The risk of developing prostate cancer increases with age [1].

Radical prostatectomy is one of the preferred methods in the treatment of localized prostate cancer. The methods for the surgery are open retropubic, laparoscopic, robot-assisted laparoscopic and perineal prostatectomy. Recently, robot-assisted radical prostatectomy is one of the most commonly used methods for the treatment of localized prostate cancer [2].

Postoperative potency and continence are significant parameters for the quality of life of patients after radical prostatectomy [3]. Urinary continence rates were found to be 33–83.8% in the first month, 52–92.3% in the third month and 89–97.9% in the first year after radical prostatectomy [4].

The patient’s body mass index (BMI), comorbidities, age, previous surgery, prostate volume, and membranous urethral length were found related with urinary incontinence [5]. In a study assessing post-prostatectomy patients for continence status, it was found that the severity of preoperative lower urinary tract symptoms, advanced age, nerve-sparing surgery and surgeon experience were identified as independent predictors for post-prostatectomy continence [6]. According to current studies, it is still difficult to predict urinary incontinence after radical prostatectomy. Apart from patient- and surgeon-related factors, mpMRI can provide important information in predicting postoperative urinary incontinence. In addition to oncological benefits, mpMRI also provides additional information about pelvic anatomy and pelvic urological organs. It was shown that the membranous urethral length measured by mpMRI has a significant impact on early postoperative continence [7].

In this study, we aimed to evaluate the preoperative patient characteristics and mpMRI findings affecting urinary continence after robot-assisted radical prostatectomy was performed.

## 2. Materials and Methods

### 2.1. Compliance with Ethical Standards

The ethical approval for the study was obtained from the Ethics Committee of Health Science University Umraniye Research and Training Hospital with approval number 0.01/262. Informed consent was obtained from all individual participants included in the study.

### 2.2. Study Design

**Figure 1:**
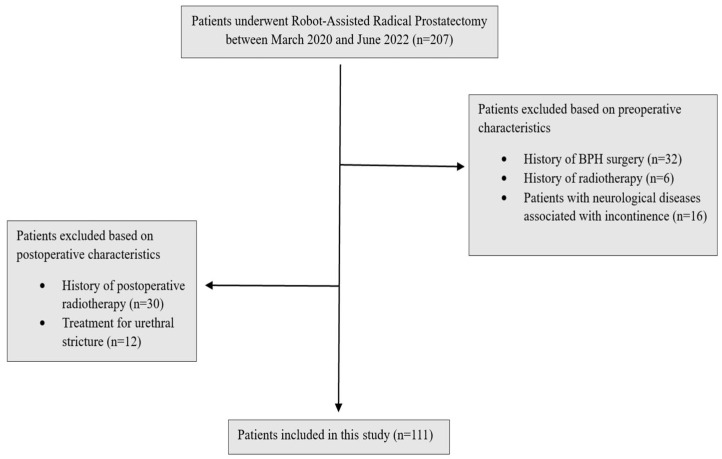
Study design.

The study was designed as a retrospective observational study. In this study, patients who underwent robotic radical prostatectomy by a single surgeon between March 2020 and June 2022 in our clinic were evaluated. The surgeon has been performing robot-assisted radical prostatectomy since 2016 and has completed more than 1600 procedures to date, ensuring a high level of surgical expertise and consistency across the cohort. The study design, and inclusion and exclusion criteria are presented in Figure 1.

**Figure 2:**
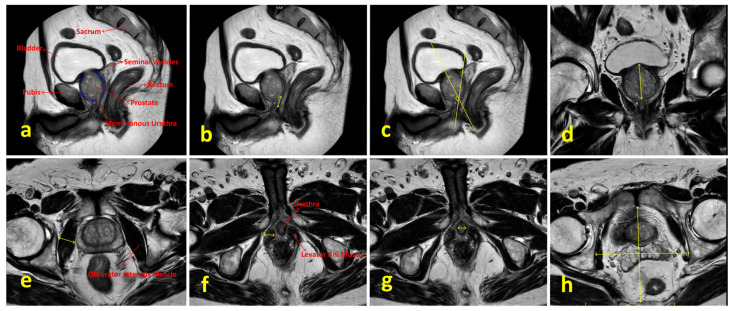
Multiparametric MRI parameters of the patients and the methods used for their measurement. (**a**) Sagittal T2-weighted image showing relevant pelvic anatomical structures. (**b**) Measurement of membranous urethral length. (**c**) Measurement of membranous urethra–prostate axis angle. (**d**) Measurement of intraprostatic urethral length in the coronal plane. (**e**) Measurement of obturator internus muscle thickness. (**f**) Visualization of the prostate apex, membranous urethra and levator ani muscle; levator ani muscle thickness is indicated by yellow arrows. (**g**) Measurement of urethral width. (**h**) Measurement of pelvic anterior–posterior and transverse diameters.

**Figure 3:**
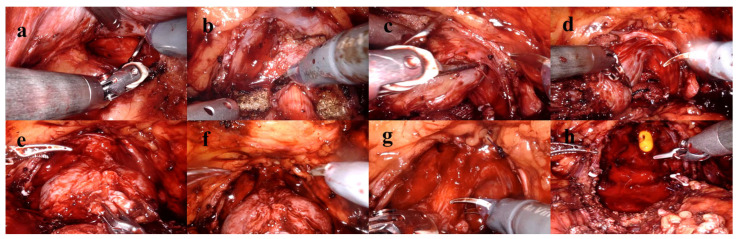
Ultrapreservation anterior-sparing technique. (**a**) Posterior dissection and seminal vesicle dissection. (**b**) Bladder neck dissection. (**c**,**d**) Dissection plane between the prostatic capsule and the neurovascular bundle. (**e**) Following transection of the dorsal venous complex. (**f**) After suturing of the dorsal venous complex. (**g**) Urethral dissection and transection. (**h**) Anatomical view prior to anastomosis.

Preoperative PSA levels, age, comorbidities, digital rectal examination findings, BMI, smoking status, lower urinary tract symptoms, sexual function, mpMRI findings, Briganti scores and surgical techniques were examined. Lower urinary tract functions were assessed using the International Prostate Symptom Score (IPSS) form, and sexual functions were evaluated using the International Index of Erectile Function (IIEF-5) form. Patients’ comorbidities were evaluated using the Charlson Comorbidity Index [8]. Preoperative prostate volume, localization and size of the tumor, presence of median lobe, membranous urethra length, membranous urethra–prostate axis angle, obturator internus muscle thickness, levator ani muscle thickness, urethral width, intraprostatic urethra length, adjacent organ invasion, pelvic anterior–posterior and transverse diameters and lymph node positivity were evaluated using mpMRI. The multiparametric MRI parameters of the patients and the measurement of these parameters are shown in Figure 2. Patients were compared in terms of postoperative continence status based on whether nerve-sparing prostatectomy was performed. The nerve-sparing technique was applied according to the previously described ultrapreservation anterior-sparing technique [9] (Figure 3).

Patients with preoperative urinary incontinence, a history of preoperative transurethral resection of prostatectomy, pelvic radiotherapy and urethral stricture, those who received postoperative radiotherapy and androgen deprivation therapy, those with neurological deficits and those aged 80 and above were excluded from the study. As part of the standard postoperative care protocol in our clinic, all patients were routinely advised to perform Kegel exercises starting from the first week after surgery. Patients’ postoperative continence status was evaluated at the first, third, sixth and twelfth months postoperatively based on daily pad usage, and EPIC-26 and ICIQ forms, without distinguishing between stress, urge or mixed subtypes. Patients who did not use any pads were classified as fully continent; those using one or two pads per day were considered moderately continent; and those using three or more pads daily were classified as incontinent. A total of 111 patients were included.

### 2.3. Statistical Analysis

Statistical analysis was performed using the most recent version (29.0.2) of IBM SPSS Statistics^®^ (IBM Corp., Armonk, NY, USA) software. The normality of the quantitative variables was assessed using histogram graphics, coefficient of variation, skewness and kurtosis values, normal Q-Q plot and detrended normal Q-Q plot graphics, and the Shapiro–Wilk test. One-way analysis of variance (ANOVA) with Bonferroni-corrected pairwise comparisons was used for comparisons among more than two groups for variables showing a normal distribution. For quantitative variables that did not follow a normal distribution, the Kruskal–Wallis test and Dunn–Bonferroni test were used for comparisons among more than two groups, while the Mann–Whitney U test was used for comparisons between two groups. The Wilcoxon Signed-Ranks test was used for within-group comparisons of quantitative variables that did not exhibit normal distribution. Pearson’s chi-square test, Fisher’s exact test and the Fisher–Freeman–Halton test were utilized for comparing qualitative data. Statistical significance was considered at *p* < 0.05.

## 3. Results

**Figure 4:**
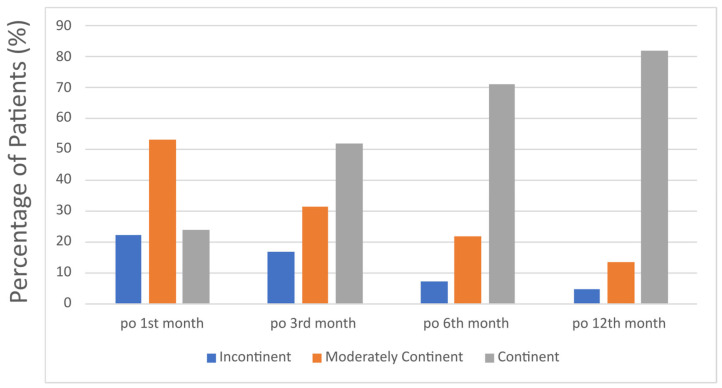
Distribution of continence status among patients at postoperative 1st, 3rd, 6th and 12th month.

A total of 111 patients were included in the study. The patients’ demographics and preoperative data are shown in Table 1. The detailed postoperative pathological characteristics of the patients are presented in Table 2. In terms of postoperative continence at the first month postoperatively, 28 patients (25%) were fully continent, 58 patients (53%) were moderately continent and 25 patients (22%) were incontinent. At the third month postoperatively, 62 patients (52%) were fully continent, 30 patients (32%) were moderately continent and 19 patients (16%) were incontinent. At the first-year follow-up, 91 patients (82%) were fully continent, 15 patients (13%) were moderately continent and 5 patients (5%) were incontinent. The continence status of patients during follow-up is shown in Figure 4.

Age, Charlson Comorbidity Index, preoperative International Index of Erectile Function (IIEF), Briganti score, and prostate volume were found to be unrelated to continence status.

The mean preoperative IPSS of the patients was 16.01 ± 9.63. At three months postoperative, patients who were fully continent showed a decrease in IPSS compared to the preoperative period. Similarly, patients who were moderately continent also experienced a decrease in IPSS scores postoperatively. Incontinent patients at three months postoperative showed a slight decrease in their IPSS compared to preoperative levels. The IPSS scores of patients, categorized by their continence levels, are summarized in Table 3.

Multiparametric MRI parameters of the patients are summarized in Table 4. At three months postoperatively, urethral width, intraprostatic urethral length, the pelvic anteroposterior and transverse diameters differed between groups.

One-way ANOVA was used to compare membranous urethral lengths, membranous urethra–prostate axis angles, obturator internus muscle thicknesses, levator ani muscle thicknesses and urethral widths according to the continence status of patients at postoperative third month. In these comparisons, patients with complete continence were found to have significantly greater urethral widths compared to those with moderate continence.

In addition to univariate comparisons, we performed a multivariable regression analysis to assess independent predictors of postoperative continence. When the clinical and mpMRI parameters were evaluated, BMI showed a statistically significant difference between fully continent and incontinent groups (*p* = 0.007), with higher BMI being associated with incontinence. Other parameters did not demonstrate statistically significant associations in the multivariable model.

Bilateral nerve-sparing surgery was performed on 76 patients, while unilateral nerve-sparing surgery was performed on 10 patients. A total of 25 patients did not undergo nerve-sparing surgery. Among the 76 patients who underwent bilateral nerve-sparing surgery, 47 were fully continent, 15 were moderately continent and 14 were incontinent at the postoperative third month follow-up. Among the 10 patients who underwent unilateral nerve-sparing surgery, 3 were fully continent, 4 were moderately continent and 3 were incontinent at the postoperative third month follow-up. Among the patients who did not undergo nerve-sparing surgery, 12 were fully continent, 11 were moderately continent and 2 were incontinent at follow-up.

## 4. Discussion

In our study, we evaluated the impact of preoperative clinical and mpMRI parameters on postoperative continence status in patients who underwent robotic radical prostatectomy and compared our findings with the literature. In a single-center study with 650 patients, it was shown that the risk of postoperative urinary incontinence increased with age [10]. In another study with 746 patients, advanced age was identified as a risk factor for urinary incontinence after radical prostatectomy [11]. In our study, no difference was found between patients’ postoperative continence status and their age. This difference may be due to the larger sample sizes in the studies of the literature. The small sample size in our study may have limited the findings between age and urinary incontinence. Therefore, we recommend that our findings be repeated with larger sample sizes.

MPMRI findings are potential data that could be used to predict postoperative continence status after radical prostatectomy. In a single-center study with 316 patients, the relationship between mpMRI findings and postoperative urinary incontinence was investigated, and shorter membranous urethral length was found to be associated with an increased risk of postoperative urinary incontinence [7]. In another study, membranous urethra length and urethral wall thickness were found to be related to postoperative urinary incontinence. According to this study, thinner urethral walls and longer membranous urethra length were associated with better postoperative continence [12]. Another study found that membranous urethra length and the pubic symphysis–prostate apex length on sagittal images were significant predictors of postoperative continence [13]. In another study evaluating 100 patients who underwent laparoscopic-assisted radical prostatectomy, membranous urethral length was measured intraoperatively using a sterile ruler. The study demonstrated that a longer membranous urethral length, along with precise apical dissection aimed at maximizing this length, was associated with improved early urinary continence [14]. A recent meta-analysis showed that a longer membranous urethral length on preoperative mpMRI reduced the risk of urinary incontinence [15]. In our study, we observed a non-significant trend toward longer membranous urethral length in incontinent patients compared to moderately continent and fully continent groups. This finding contrasts with the majority of previous studies, which have consistently reported shorter membranous urethral length as a predictor of worse postoperative continence outcomes. We believe this discrepancy may be attributed to the retrospective design of our study and the relatively small sample size, both of which may have limited the statistical power and increased the likelihood of random variation. While our data did not support membranous urethral length as a significant predictive factor for continence recovery, further prospective studies with larger sample sizes and standardized methodologies are warranted to better elucidate this relationship. One of the notable findings of our study was the significant association between pelvic diameters and postoperative urinary continence. Specifically, patients who experienced postoperative urinary incontinence were found to have narrower pelvic anteroposterior and transverse diameters. This contrasts with a previous study involving 50 patients, which did not demonstrate a significant relationship between pelvic dimensions and postoperative continence outcomes [16]. A wider pelvic diameter may facilitate easier perioperative dissection, reduce anastomosis time, and allow for better preservation of the pelvic floor and periprostatic supporting tissues that are crucial for maintaining urinary continence following prostate surgery. Furthermore, we also observed that at the third postoperative month, urethral width was significantly greater in patients with complete continence compared to those with moderate continence. These findings suggest that, alongside membranous urethral length, both pelvic dimensions and urethral width may serve as valuable predictors of postoperative urinary continence. Nevertheless, further prospective and larger-scale studies are warranted to validate these associations and to better understand the underlying mechanisms involved.

The patients participating in our study were evaluated for lower urinary tract symptoms using the IPSS form, both preoperatively and postoperatively. Patients who experienced postoperative incontinence had significantly higher preoperative IPSS scores. When comparing postoperative IPSS scores, the IPSS of continent cases was significantly lower compared to cases with moderate continence and incontinence. Although IPSS primarily reflects obstructive symptoms, it also includes items related to frequency, urgency, nocturia and quality of life, which provide valuable information regarding nonobstructive LUTS in the postoperative setting. Therefore, we consider IPSS to be a meaningful tool for evaluating changes in overall urinary function following surgery. Higher preoperative IPSS scores were associated with an increased risk of postoperative urinary incontinence. Another study evaluating post-prostatectomy incontinence found an association between preoperative severe lower urinary tract symptoms and the risk of postoperative incontinence [6].

In the study conducted by Collette and colleagues, age, BMI, preoperative IPSS, surgeon experience, use of the nerve-sparing technique, prostate volume, and the patient’s ASA (American Society of Anesthesiologists) score were found to be associated with the risk of postoperative incontinence [6]. In line with their findings, our multivariable regression analysis also identified higher BMI as a significant independent predictor of incontinence. We did not observe a statistically significant relationship between prostate volume and continence status in our cohort. This difference may have resulted from the relatively small sample size in our study, which could have limited the statistical power to detect weaker associations between prostate volume and postoperative continence outcomes.

When comparing patients based on the application of nerve-sparing surgery, we observed a higher rate of nerve-sparing technique usage in cases with moderate continence compared to incontinent cases. In cases with complete continence, however, the rate of bilateral nerve-sparing surgery was higher compared to cases with moderate continence.

Although our study provides valuable insights into several parameters, it does have limitations. One of the main limitations is its retrospective nature, along with the small sample size. Despite the fact that robotic surgery has been performed for many years and all cases were carried out by a single expert surgeon, the fact that this is a single-center study can also be considered a limitation. Another important limitation of this study is the absence of a control group. The lack of a control group makes it difficult to compare the outcomes of robot-assisted radical prostatectomy with other surgical techniques such as open or laparoscopic radical prostatectomy and may limit the generalizability of our findings to broader patient populations.

One of the strengths of our study is the close follow-up of our patients for continence. Another strength is the evaluation of MRI parameters in addition to preoperative and perioperative findings of the patients. There are studies in the literature evaluating the relationship between preoperative mpMRI and the risk of postoperative urinary incontinence [7,12,17]. There are also studies evaluating the relationship between patient-related parameters and the risk of postoperative urinary incontinence in the literature [6,17,18]. In our study, we evaluated patient-related parameters including age, BMI, comorbidity score, smoking status, preoperative IIEF, IPSS, and rectal examination findings, as well as MRI findings.

## 5. Conclusions

In conclusion, our findings suggest that both clinical and mpMRI parameters may have potential value in estimating postoperative urinary continence following robotic radical prostatectomy. While age was not associated with continence status in our cohort, factors such as BMI, pelvic diameters, membranous urethral length, urethral width and preoperative IPSS scores appeared to be associated with continence outcomes. These preliminary results indicate that a more comprehensive preoperative evaluation including mpMRI measurements and symptom scores could contribute to better continence outcomes. However, given the retrospective nature of this study these findings should be interpreted with caution. Further prospective, multicenter studies with larger patient populations are warranted to confirm and expand upon these observations.

## Figures and Tables

**Table 1:** Demographics and preoperative patient data.

Demographics		
Age	61.5 ± 6.7	mean ± SD
BMI	27.7 (20.1–39)	median (min–max)
Charlson Comorbidity Index	4 (2–6)	median (min–max)
Prostate Specific Antigen (ng/mL)	6.5 (2.44–24)	median (min–max)
IIEF	16 (5–25)	median (min–max)
IPSS	15 (1–35)	median (min–max)
Briganti Score	3.1 (1–45.9)	median (min–max)
Prostate Volume (ml)	50 (12–238)	median (min–max)

BMI: Body mass index; IIEF: International Index of Erectile Function; IPSS: International Prostate Symptom Score; ml: milliliter; ng/mL: nanogram per milliliter.

**Table 2:** Final pathological outcomes of the study cohort.

Final Pathological Outcomes			
pT Stage	pN Stage	ISUP Grade	Surgical Margin
T2a (*n* = 20) T2b (*n* = 18) T2c (*n* = 32) T3a (*n* = 32) T3b (*n* = 9)	Nx (*n* = 71) N0 (*n* = 37) N1 (*n* = 3)	Grade Group 1 (*n* = 15) Grade Group 2 (*n* = 84) Grade Group 3 (*n* = 10) Grade Group 4 (*n* = 0) Grade Group 5 (*n* = 2)	R0 (*n* = 88) R1 (*n* = 23)

pT: pathological tumor stage; pN: pathological nodal status; R0: negative surgical margin; R1: positive surgical margin; ISUP Grade: International Society of Urological Pathology Grade.

**Table 3:** Evaluation of IPSS measurements according to continence levels postoperative 3rd month.

	Total Continent (*n* = 62)	Moderately Continent (*n* = 30)	Incontinent (*n* = 19)	*p*
Preoperative IPSS	9 (1–18)	18 (2–36)	17 (1–34)	0.033 ^a^
Postoperative 3rd month IPSS	5 (0–21)	8 (0–16)	9 (5–13)	0.001 ^a^
Postoperative 1st year IPSS	3 (0–26)	3 (0–12)	6 (1–11)	0.007 ^a^
Difference between preoperative and postoperative 3rd IPSS	4 (12–32)	10 (6–32)	9 (6–29)	0.369 ^a^
*p*	0.002 ^b^	0.001 ^b^	0.001 ^b^	

Kruskal–Wallis Test (^a^); Wilcoxon Signed-Ranks Test (^b^).

**Table 4:** Multiparametric MRI measurement outcomes according to continence levels at postoperative 3rd month.

	Total Continent (*n* = 62)	Moderately Continent (*n* = 30)	Incontinent (*n* = 19)	*p*
Membranous urethral length (mm)	14.5 (10.6–24.8)	15.6 (10.6–21.4)	15.8 (11.8–21.4)	0.101 ^c^
Membranous urethra–prostate axis angle	56.6 (33.6–77.5)	55.2 (32.7–86.6)	59.4 (44.3–76.4)	0.531 ^c^
Obturator internus muscle thickness (mm)	19.9 (13.4–25.9)	20.3 (15.4–27.4)	20.3 (13–27.7)	0.900 ^c^
Levator ani muscle thickness (mm)	9.9 (6.3–14.9)	10.5 (6.8–12.2)	10.4 (8.5–12.4)	0.825 ^c^
Urethral width (mm)	10.8 (6.7–13.9)	9.6 (7–12.4)	10.1 (8.3–14.1)	0.012 ^c^
Intraprostatic urethral Length (mm)	32.4 (20.7–56.6)	36.1 (19–57.6)	35.2 (29–52.5)	0.079 ^a^
Pelvis AP diameter (mm)	124.9 (101.5–141.6)	128.4 (119–139.8)	121.2 (80.5–134.7)	0.033 ^a^
Pelvis transverse diameter (mm)	117.4 (96.5–146.1)	121.2 (103.1–151.3)	114 (96.8–132.8)	0.002 ^a^

One-way ANOVA (^c^); Kruskal–Wallis Test (^a^).

## Data Availability

All data analyzed in the present study are included in this article.

## Abbreviations

The following abbreviations are used in this manuscript:

AP	Anterior–Posterior
ASA	American Society of Anesthesiologists
BMI	Body Mass Index
IPSS	International Prostate Symptom Score
IIEF	International Index of Erectile Function
mpMRI	Multiparametric Magnetic Resonance Imaging
PCa	Prostate Cancer
PSA	Prostate Specific Antigen
RARP	Robot-Assisted Radical Prostatectomy
